# Knowledge on HBV vaccine and vaccination status among health care workers of Hawassa University Comprehensive Specialized Hospital, Hawassa, southern Ethiopia: a cross sectional study

**DOI:** 10.1186/s13104-018-4023-0

**Published:** 2018-12-20

**Authors:** Asres Bedaso, Bereket Duko, Remla Fedlu

**Affiliations:** 1 College of Medicine and Health Sciences, School of Nursing, Hawassa University, Hawassa, SNNPR Ethiopia; 2 Hawassa University Comprehensive Specialized Hospital, Hawassa, SNNPR Ethiopia

**Keywords:** HBV, Knowledge, Vaccination status, Health care worker

## Abstract

**Objectives:**

The study was conducted to assess the knowledge on HBV vaccine and vaccination status among health care workers of Hawassa University Comprehensive Specialized Hospital.

**Result:**

From the total 258 questionnaire prepared for the study participant, data was collected from 241 participants making the response rate of 93.4%. Regarding socio-demographic characteristics of respondents 98 (40.7%), and 159 (66%) were females and Bachelor of Science graduates respectively. Only 73 (30.3%) respondents reported that they are vaccinated for hepatitis B vaccine. But only 16 (21.9%) received three doses of Hepatitis B vaccine, which was 6.6% of the total health care workers. More than half 146 (60.6%) of the respondents had good knowledge about hepatitis B virus infection and 120 (49.8%) had good knowledge about hepatitis B vaccine. Regarding knowledge about hepatitis B virus infection prevention and control methods, 131 (28.5%) of the respondents have good knowledge.

**Electronic supplementary material:**

The online version of this article (10.1186/s13104-018-4023-0) contains supplementary material, which is available to authorized users.

## Introduction

Hepatitis is an inflammation of the liver due to viral infections and there are groups of viruses that affect the liver. Hepatitis B virus (HBV) infection threatens the health of populations across the globe. It is a DNA virus that cause viral hepatitis and can lead to liver cirrhosis and hepatocellular carcinoma [1, 2].

An estimated 240 million people are chronically infected and more than 780,000 people die every year due to complications of hepatitis B including cirrhosis and liver cancer [3]. The spectrum of clinical manifestations of hepatitis B virus (HBV) infection varies in both acute and chronic disease. During the acute phase, manifestations range from sub clinical or anicteric hepatitis to icteric hepatitis; during the chronic phase manifestations range from asymptomatic carrier state to chronic hepatitis, cirrhosis and hepatocellular carcinoma [4].

The World Health Organization estimates that about 2 million health care workers face occupational exposure to HBV each year and 90% of the infections that result from these exposures are in low-income countries, especially those in sub-Saharan Africa [5].

Liver diseases are common in Africa and account for high morbidity and mortality. Reports from hospital based studies show that about 12% of medical admissions and more than 20% of hospital mortality in many parts of Africa were due to acute viral hepatitis, chronic hepatitis, cirrhosis and hepatocellular carcinoma. Chronic carrier rates among the general population in Nigeria ranged from about 12% to 48.7% in different states and cities [6].

Ethiopia, being part of sub-Saharan region, is ranked as an area with medium to high endemicity for HBV infection, based on previous population surveys [7]. Ethiopia, which has significant Hepatitis B transmission in both children and adults displays around 6% to 12% hepatitis B surface antigen (HBs Ag) prevalence. Liver disease is known to be a significant cause of hospital admission in Ethiopia although the etiology of this disease is not well defined. Acute viral hepatitis, chronic hepatitis and cirrhosis of the liver account for 12% of hospital admissions and 31% of mortality on medical wards in Ethiopia [8].

Hepatitis B infection is a major public health problem in Ethiopia and health care workers are also at increased risk of acquiring hepatitis B infection due to occupational exposure [9, 10]. Therefore, the aim of this study is to assess the knowledge about hepatitis B vaccine and vaccination status among health care workers of Hawassa University Comprehensive Specialized Hospital.

## Main text

### Methods

#### Study design, study area and period

Facility based cross-sectional study design was conducted from Feb 3, 2017 to May 29, 2017, among health care workers of Hawassa University Comprehensive Specialized Hospital. Hawassa University Comprehensive Specialized Hospital is found in Hawassa city, 273 km far south from Addis Ababa. The hospital started giving service since 2005 and serves for more than 18 million people of the catchment area. The hospital had more than 400 beds for inpatient service and also gives different outpatient services.

#### Sample size determination and sampling procedure

Sample size of this study was determined using single population proportion formula by considering 95% level of confidence, 5% margin of error and proportion of health care workers (HCWs) who were knowledgeable about hepatitis B vaccine 62% is considered [17]. Since the sampling frame is less than 10,000, correction formula was used. After adding 10% none response rate, the final sample size was 258. Considering list of health care workers as a sampling frame, which we get from the hospital human resource development office, Simple random sampling technique was used to select eligible study participants from the hospital.

#### Source and study population

All health care workers working in Hawassa University Comprehensive Specialized Hospital were the source population. Health care workers working in Hawassa University Comprehensive Specialized Hospital who are available during data collection period were considered as source population.

#### Data collection technique and instrument

Self-administered technique was used to collect data from selected study participants. The questionnaire had three parts; the first part contains socio-demographic characteristics of HCWs, the second part was knowledge of HCWs on hepatitis B vaccine. Level of knowledge of HCWs was categorized based on the mean value; those who scored below the mean were classified as less knowledgeable and those who scored above the mean were classified knowledgeable. The third part was about hepatitis B vaccine status of HCWs. Before data collection pretest was done at Adare Hospital to test the reliability of the tool on 5% of the sample size.

#### Data analysis

The collected data was entered and analyzed using Statistical Package for Social Sciences (SPSS 20). Then, data was edited, coded, cleaned and some consistency checks were made to have quality data.

### Result

From the total 258 questionnaires prepared for the study participant 241 responded and include in the analysis making the response rate of 93.4%. 98 (40.7%) of respondents were females and 117 (48.5%), 45 (18.7%) and 33 (13.7%) of the respondents were nurses, physician and midwifes by their occupation respectively. Regarding educational level of the respondent’s; majority 159 (66%) of them had Bachelor of Science degree (Table 1).

**Table 1:** Socio demographic characteristics of health care workers at Hawassa University Comprehensive Specialized Hospital, Hawassa, SNNPR, Ethiopia, 2017

Socio demographic characteristic	Frequency	Percent %
Sex		
Male	143	59.3
Female	98	40.7
Occupation		
Physician	45	18.7
Nurse	117	48.5
Midwife	33	13.7
Laboratory technician	20	8.3
Pharmacists	5	2.1
Dentist	1	0.4
Medical specialist	10	4.1
Surgical specialist	8	3.3
Other	2	0.8
Marital status		
Single	149	61.8
Married	89	36.9
Windowed	1	0.4
Divorced	2	0.8
Department		
Outpatient department	22	9.1
Emergency room	45	18.7
Operation room	32	13.3
Delivery room	27	11.2
Ward	98	40.7
Other	17	6.9
Educational level		
Diploma	46	19.1
Degree (B.Sc.)	159	66
Master degree	10	4.1
Other	26	10.8
Working duration (years)		
< 5	203	84.2
≥ 5	38	15.8
Vaccination status		
Yes	73	30.3
No	168	69.7
No of HBV dose taken		
One time	34	46.6
Two time	23	31.5
Three time	16	21.9

#### History of ever occupational exposure to conditions that predisposed to HBV infection

Regarding exposure to conditions that might lead to HBV infection 153 (63.5%) of the participant reported that they had history of exposure to blood or body fluids on intact skin (Table 2).

**Table 2:** History of ever occupational exposure to conditions that predisposed to HBV infection of health care workers at Hawassa University Comprehensive Specialized Hospital, Hawassa, SNNPR, Ethiopia, 2017

Question	Yes, number (%)
History of exposure to blood or body fluids on intact skin	153 (63.5)
History of splash of blood or body fluids to eye or mouth	81 (33.6)
History of splash of blood on cuts or unprotected skin	57 (23.7)

#### Knowledge about hepatitis B infection

Study participants were asked about their knowledge about hepatitis B infection using 8 item tool. The minimum and the maximum score to the question were 1 and 8 respectively. The mean knowledge score for HBV infection was 6.63 (SD ± 0.9).

Finally the overall category of the study participants showed that more than half 146 (60.6%) of the respondents had good knowledge about hepatitis B infection (Table 3).

**Table 3:** Knowledge of health care workers about hepatitis B infection at Hawassa University Comprehensive Specialized Hospital, Hawassa, SNNPR, Ethiopia, 2017

Knowledge question about hepatitis B infection	True, N (%)
One can get hepatitis B infection through needle stick injury	235 (97.5)
Hepatitis B infection can be prevented by vaccination	233 (96.7)
Hepatitis B virus can be found in semen or vaginal fluid of infected person	225 (93.4)
Hepatitis B infected person may be asymptomatic for long time	218 (90.5)
Every person exposed to hepatitis B virus will develop acute hepatitis immediately	81 (33.6)
Hepatitis B virus is highly infectious	226 (93.8)
Only small proportion of the world population is infected with hepatitis B virus	79 (32.8)
Hepatitis B virus mainly affects live	217 (90)

#### Knowledge of hepatitis B infection prevention and control measures

Respondents were asked 14 questions about hepatitis B infection. The maximum and minimum scores were 14 and 7 respectively. The mean score of the respondents about hepatitis B infection prevention and control method was 12.29 with standard deviation of 1.7 and 131 (28.5%) of the respondents scored above the mean (Additional file 1).

#### Knowledge of hepatitis B vaccine

Respondents were asked 12 item questions to assess their knowledge about Hepatitis B vaccine. The maximum and minimum scores for the questions were 11 and 3 respectively. The mean knowledge score for hepatitis B vaccine was 7.54 with standard deviation of 1.74. Overall, the assessment of knowledge of respondents about Hepatitis B vaccine indicates 120 (49.8%) had good knowledge about the hepatitis B vaccine (Additional file 2).

### Discussion

Health care workers are at risk of acquiring blood born disease including HBV due to occupational exposure to blood and body fluids [9, 10]. From 241 Health Care Workers (HCWs) who responded the question whether they had history of occupational exposure to blood or body fluid on intact skin or not, 153 (63.5%) reported history of occupational exposure. This indicates there is high level of occupational exposure to hepatitis B and other blood borne pathogens.

This study also assessed that 30.3% of study participants were vaccinated and Out of the vaccinated 73 respondents, only 16 (21.9%) were fully vaccinated. This finding is higher than the study conducted in Bahirdar [17] where only 10% were vaccinated and out of which only 5.4% were fully vaccinated. The result in the current study is very low in comparison to other studies conducted in Burkina Faso [16] where 58.6% were partially and 10.9% were fully vaccinated, Nigeria [12] 65% vaccinated, Syria [13] 76.6%, Morocco [11], 55%, north India [15] 60% partially and 40% fully vaccinated, Greece [14] 57.1% were vaccinated and USA [13] 75% were fully vaccinated. The variation might be due to the difference in health system of the countries, availability and accessibility of the vaccine.

The finding in the current study indicates that 146 (60.6%) of the study participants had good knowledge about HBV infection which is lower than the finding of the study conducted in Nigerian Lagos State [18] where 70.2% of study participant were knowledgeable about HBV infection. But the finding of this study is higher than the study conducted in Addis Ababa [8] where 55.1% of study participants had good knowledge about HBV infection. The variation might be due to the difference in health system.

Regarding knowledge about hepatitis B vaccine, in this study 40.9% of study participants had good knowledge about Hepatitis B vaccine which is lower than the study conducted in Nigeria [12] where 77% of study participants were knowledgeable about the vaccine. The difference might be due to the variation among health care workers, where in some countries training and orientation was given for HCWs.

### Conclusion

More than half of study participants found to have poor knowledge about HBV. This study also found that the vaccination status of respondents to be very low and when we consider the long term severity of the disease and the increased exposure risk of health care workers, it is better to avail and provide hepatitis B vaccine for health care workers as a work place safety.

## Limitation

The cross sectional nature of the present study prevents any causal inferences.

## Additional files

**Additional file 1.** Knowledge of health care workers about hepatitis B infection prevention and control measures at Hawassa University Comprehensive Specialized Hospital, Hawassa, SNNPR, Ethiopia, 2017.

**Additional file 2.** Knowledge of health care workers about hepatitis B vaccine at Hawassa University Comprehensive Specialized Hospital, Hawassa, SNNPR, Ethiopia, 2017.

## Abbreviations

DNA: deoxyribonucleic acid
HBs Ag: hepatitis B surface antigen
HBV: hepatitis B vaccine
HCWs: Health Care Workers
SPSS: Statistical Package for Social Science
