# Supplementary material for: Knowledge on HBV vaccine and vaccination status among health care workers of Hawassa University Comprehensive Specialized Hospital, Hawassa, southern Ethiopia: a cross sectional study
Source: BMC Res Notes. 2018 Dec 20;11:912. doi: 10.1186/s13104-018-4023-0 (PMC6302467; doi:10.1186/s13104-018-4023-0)
Supplement: Supplementary file 1 — Additional file 1. Knowledge of health care workers about hepatitis B infection prevention and control measures at Hawassa University Comprehensive Specialized Hospital, Hawassa, SNNPR, Ethiopia, 2017. [file 13104_2018_4023_MOESM1_ESM.docx]

Additional file 1. Knowledge of health care workers about hepatitis B infection prevention and control measures at Hawassa university comprehensive specialized hospital, Hawassa, SNNPR, Ethiopia, 2017

| Knowledge questions | Yes N (%) | | |
| --- | --- | --- | --- |
| Hepatitis virus can be transmitted from one person to the other through | | | |
| Sharps injury | | 236(97.9) | |
| Blood donation from infected person | | 237(98.3) | |
| Sexual intercourse with infected person | | 230(95.4) | |
| From mother to child during pregnancy | | 224(92.9) | |
| Feco oral | | 100(41.5) | |
| Polluted water | | 51(21.2) | |
| Transmission of hepatitis B infection can be prevented by | | | |
| Vaccination | | | 235(97.5) |
| Proper disposal sharp materials | | | 236(97.9) |
| Avoiding multiple sexual Partner | | | 231(95.9) |
| Avoiding drinking contaminated water | | | 62(25.7) |
| Avoiding uncooked food | | | 50(20.7) |
| Using glove | | | 234(97.1) |
| There is a higher risk of Hepatitis B than HIV transmission through needle stick injury | | | 229(95) |
| The symptoms of hepatitis B viral infection appear within few days always after the entrance of Hepatitis B virus to the body | | | 71(29.5) |
| Have you ever taken training on infection prevention? | | | 69(28.6) |
