# Supplementary material for: Knowledge on HBV vaccine and vaccination status among health care workers of Hawassa University Comprehensive Specialized Hospital, Hawassa, southern Ethiopia: a cross sectional study
Source: BMC Res Notes. 2018 Dec 20;11:912. doi: 10.1186/s13104-018-4023-0 (PMC6302467; doi:10.1186/s13104-018-4023-0)
Supplement: Supplementary file 2 — Additional file 2. Knowledge of health care workers about hepatitis B vaccine at Hawassa University Comprehensive Specialized Hospital, Hawassa, SNNPR, Ethiopia, 2017. [file 13104_2018_4023_MOESM2_ESM.docx]

Additional file 2. Knowledge of health care workers about hepatitis B vaccine at Hawassa university comprehensive specialized hospital, Hawassa, SNNPR, Ethiopia, 2017

| Knowledge question about HB vaccine | True N (%) |
| --- | --- |
| There is effective vaccine to prevent hepatitis B infection | 221(91.7) |
| Hepatitis B vaccine can be given as post-exposure prophylaxis | 139(57.7) |
| Hepatitis B vaccine is contra indicated for immune compromised patients | 173(71.8) |
| Hepatitis B vaccine is effective to treat patients with acute hepatitis B infection | 56(23.2) |
| Hepatitis B vaccine is highly effective in preventing hepatitis B infection if given within 48 hours after exposure | 154(63.9) |
| Hepatitis B vaccine should be given to health care workers as part of work place safety | 204(84.6) |
| Full course of hepatitis B vaccine may give lifelong immunity but for Health professionals, one further booster after 5 years of the first dose is recommended | 149(61.8) |
| After taking full dose vaccination of hepatitis B, there is no need for a blood test to confirm immunity against hepatitis B | 72(29.9) |
| Full dose hepatitis B vaccine provides 100% protection for 90% of adults | 145(60.2) |
| Full dose hepatitis B vaccine protects against HBV for at least 15 years | 141(58.5) |
| Hepatitis B vaccine causes problems if given to people who are already immune | 111(46.1) |
| Hepatitis B vaccine is recommended for all health care workers | 228(94.6) |
